# Predicting the Development of Surgery-Related Pressure Injury Using a Machine Learning Algorithm Model

**DOI:** 10.1097/JNR.0000000000000411

**Published:** 2020-12-21

**Authors:** Ji-Yu CAI, Man-Li ZHA, Yi-Ping SONG, Hong-Lin CHEN

**Affiliations:** 1 BSN, Graduate Student, School of Nursing, Nantong University, Nantong City, Jiangsu, People’s Republic of China; 2 MS, Associate Professor, School of Nursing, Nantong University, Nantong City, Jiangsu, People’s Republic of China.

**Keywords:** surgery-related pressure injury, machine learning, risk assessment, cardiovascular surgery

## Abstract

**Background:**

Surgery-related pressure injury (SRPI) is a serious problem in patients who undergo cardiovascular surgery. Identifying patients at a high risk of SRPI is important for clinicians to recognize and prevent it expeditiously. Machine learning (ML) has been widely used in the field of healthcare and is well suited to predictive analysis.

**Purpose:**

The aim of this study was to develop an ML-based predictive model for SRPI in patients undergoing cardiovascular surgery.

**Methods:**

This secondary analysis of data was based on a single-center, prospective cohort analysis of 149 patients who underwent cardiovascular surgery. Data were collected from a 1,000-bed university-affiliated hospital. We developed the ML model using the XGBoost algorithm for SRPI prediction in patients undergoing cardiovascular surgery based on major potential risk factors. Model performance was tested using a receiver operating characteristic curve and the C-index.

**Results:**

Of the sample of 149 patients, SRPI developed in 37, an incidence rate of 24.8%. The five most important predictors included duration of surgery, patient weight, duration of the cardiopulmonary bypass procedure, patient age, and disease category. The ML model had an area under the receiver operating characteristic curve of 0.806, which indicates that the ML model has a moderate prediction value for SRPI.

**Conclusions:**

Applying ML to clinical data may be a reliable approach to the assessment of the risk of SRPI in patients undergoing cardiovascular surgical procedures. Future studies may deploy the ML model in the clinic and focus on applying targeted interventions for SRPI and related diseases.

## Introduction

Pressure injury is generally defined as localized damage to the skin and underlying soft tissue, usually because of the location of the skin and tissue over a bony prominence or of the use of a medical or other device (Xiong et al., 2019). During surgery, patients are affected by procedure-related factors such as perioperative fasting, liquid fasting, postanesthesia compulsive position, and disinfectant-induced damp skin (Gao et al., 2018). Therefore, patients face an elevated risk of experiencing a surgery-related pressure injury (SRPI). A meta-analysis reported a general prevalence of SRPI of 18.96% (95% CI [15.3, 22.6]) among patients (Shafipour et al., 2016). An incidence rate of SRPI ranging from 0.3% to 57.4% and 18% among patients who underwent cardiovascular surgery was identified in a systematic review (95% CI [14.0, 22.0]; Chen et al., 2012). Pressure injury is an important safety indicator in healthcare systems. Pressure injuries not only adversely affect quality of life but also drain resources from healthcare systems worldwide (Girouard et al., 2008; Liao et al., 2018).

Commonly identified risk factors for SRPI include surgical positioning, type of anesthesia, duration of surgery, extracorporeal circulation, and pressure from internal retractors or from operating room staff (Campbell, 1989; Papantonio et al., 1994; J. Walsh, 1993). Evidence from clinical trials suggests that pressure injury is preventable in today's modern healthcare environment (Thomson & Brooks, 1999). Assessing the risk of pressure injury is recommended in clinical nursing care. Unfortunately, although some risk assessment tools for SRPI have been developed, they have limitations. Although the Braden Scale is a validated and widely used instrument for assessing pressure injury risk, this scale was developed for use in other care settings. The validity and reliability of using the Braden Scale to assess pressure injury development have been established in a variety of patient care settings. However, the results of a meta-analysis revealed that the Braden Scale had a low predictive value for SPRI development in patients who underwent surgery (He et al., 2012). Alternatively, the modified Norton scale has been used frequently in German hospitals. However, the sensitivity and specificity of this scale are 41% and 88%, respectively (Feuchtinger et al., 2007). The Waterlow score is another widely used instrument for predicting pressure injury development. The studies indicated that this scale, although suitable for predicting postoperative morbidity and mortality in surgical patients, did not predict the likelihood of SRPI formation (Thorn et al., 2013). These traditional risk assessment scales are based on clinical experience dating from the 1970s and 1980s and lack sufficient data and evidence-based support.

Machine learning (ML), a recently developed field of smart technology, is increasingly being applied in the construction of predictive models. Incorporating ML improves the performance of predictive models and does not require explicit programming or manual guidance (Ethem, 2004; Marsland, 2009). Thus, ML is now widely recognized as an effective resource in medicine and healthcare. In a previous study, an ML-based predictive model constructed for urinary tract infections in the emergency department was superior to existing predictive models (Taylor et al., 2018). Another ML algorithm, XGBoost, was used to construct a risk prediction model for incident essential hypertension and achieved satisfactory predictive accuracy (Ye et al., 2018). Applying ML to longitudinal clinical data has provided a scalable tool for expanding screening for risk of nonfatal suicide attempts in adolescents (C. G. Walsh et al., 2018). Furthermore, ML techniques effectively and efficiently use large amounts of clinical data and are well suited for use in predicting SRPI.

The aims of this study were to integrate ML information with clinical information to predict SRPI risk among patients and to validate the validity of the developed ML predictive model as a reference for future studies.

## Methods

### Sample

This study adopted a secondary analysis approach using data from a prospective study of patients who had consecutive cardiac surgeries that was conducted to predict the incidence of SRPI using ML (Lu et al., 2017). The sample consisted of data on patients who underwent cardiac surgery and aortic surgery at a teaching hospital between January 2015 and December 2015. The inclusion criteria included all patients, regardless of age, with a pressure injury at the time of admission and before surgery. This study was approved by the ethics committee of the School of Nursing, Nantong University (Approval No. 2018056) in China.

### Data Collection

Data for each patient were obtained from the original health records. A wide range of relevant predictors noted in prior studies, including demographic characteristics, SRPI information, and corticosteroid information, among others, were considered. Demographic characteristics included age, gender, weight, and disease category; SRPI information included number of ulcers, ulcer severity as determined using the National Pressure Ulcer Advisory Panel classification (Choi et al., 2016), anatomical location, and outcome; corticosteroid information included administration, type of drug used, drug dosage, and frequency of drug administration; and risk factors included use of vasoactive drugs, experiencing hypotensive periods, hemoglobin level, albumin level, and use of corticosteroids (Feuchtinger et al., 2005).

### Model Construction

Models were constructed using all of the variables collected, and descriptive statistics were used to compare the baseline characteristics and outcomes. Univariate chi-square tests and *t* tests were used to compare categorical variables and continuous variables, respectively. *P* values (< .10) were deemed significant. The prospective data were used to construct the prediction model. XGBoost, an algorithm in ML, was used to generate predictive estimates on the basis of features retained in the univariate analysis (Plagnol et al., 2012). XGBoost is a novel, sparsity-aware algorithm used in conditions of sparse data and weighted quantile sketch for approximate tree learning. It is designed to improve the speed and performance of gradient-boosted decision trees. The final predictive estimate is calculated by summing the scores in the corresponding leaves of each tree (Ye et al., 2018). XGBoost adds an estimator to provide a better approximation. At each iteration, a new prediction model is built, and each model learns to correct the previous stage model. XGBoost does not require linear features or linear interactions between features and thus is a significantly better classifier than other algorithms. The model usually refers to the mathematical structure of how to make prediction-dependent variables given the independent variables. This algorithm has been recognized as having good accuracy, flexibility, and speed.

### Model Evaluation

The performance of the model was evaluated based on the mixture matrix. The primary indicator of this prediction model was the area under the curve (AUC) of the receiver operating characteristic. The secondary indicators were sensitivity and accuracy with 95% CI. Most classification models have an AUC between 0.5 and 1, a random classifier has an AUC of 0.5, and a perfect classifier has an AUC of 1 (Qiao et al., 2018). The definition of sensitivity is the proportion of positive results out of the number of samples that were positive and the proportion of negative results out of the number of samples that were negative (Taylor et al., 2018).

## Results

### Patient Characteristics

This study included data from 149 patients, with ages ranging from 4 to 77 years. Seventy-nine patients were male, and 70 were female. In terms of disease category, 35 of the patients had congenital heart disease, 85 had valvular heart disease, 20 had coronary heart disease, and nine had macrovascular disease. Selected patient characteristics stratified by cardiovascular surgical patients are shown in Table 1.

**Table 1.** Baseline Patient Characteristics (*N* = 149)

Characteristic	Without SRPI (*n* = 112)		With SRPI (*n* = 37)		*p*
	*n*	%	*n*	%	
Gender					.815
Male	60	53.6	19	51.4	
Female	52	46.4	18	48.6	
Disease category					.074
Congenital heart disease	32	28.6	3	8.1	
Valvular heart disease	61	54.5	24	64.9	
Coronary artery disease	13	11.6	7	18.9	
Thoracic aortic aneurysms	6	5.4	3	8.1	
Vasoactive agents intraoperatively					.768
Yes	30	26.8	9	24.3	
No	82	73.2	28	75.7	
Vasoactive agents postoperatively					.737
Yes	76	67.9	24	64.9	
No	36	32.1	13	35.1	
Corticosteroids perioperative					.018
Yes	5	4.5	6	16.2	
No	107	95.5	31	83.8	
	*M*	*SD*	*M*	*SD*	*p*
Age (years)	48.2	18.3	54.7	15.0	.053
Weight (kilograms)	59.8	15.3	59.1	15.4	.805
Surgery duration (minutes)	221.7	85.8	263.6	93.0	.013
CPB duration (minutes)	48.9	23.1	48.9	23.1	.996

***Note.*** SRPI = surgery-related pressure injury; CPB = cardiopulmonary bypass.

### Model Performance

The ML tool XGBoost was selected to construct the SRPI prediction model. The outcome evaluation index was the occurrence of SRPI. A score of 1 would be assigned to patients who developed SRPI, and a score of 0 would be assigned otherwise. We evaluated the SRPI prediction model in the form of confusion matrices, with sensitivity, specificity, and the Youden Index. In the prediction model, three patients were correctly predicted as positive for SRPI, and 34 were erroneously predicted as negative. One hundred twelve patients with no SRPI were correctly predicted as negative. The values of sensitivity and specificity were, respectively, 8.11% and 100%. The Youden Index was derived using the threshold at which the sum of sensitivity and specificity achieves the highest value. The value of Youden Index was calculated as 0.081.

Positive predictive value and negative predictive value were used to describe the performance of the SRPI prediction model. The positive predictive value was 100%, indicating that 100% of patients who developed SRPI were predicted to do so. The negative predictive value was 76.71%, indicating that 76.71% of patients with no SRPI were predicted to do so.

**Figure 1.**
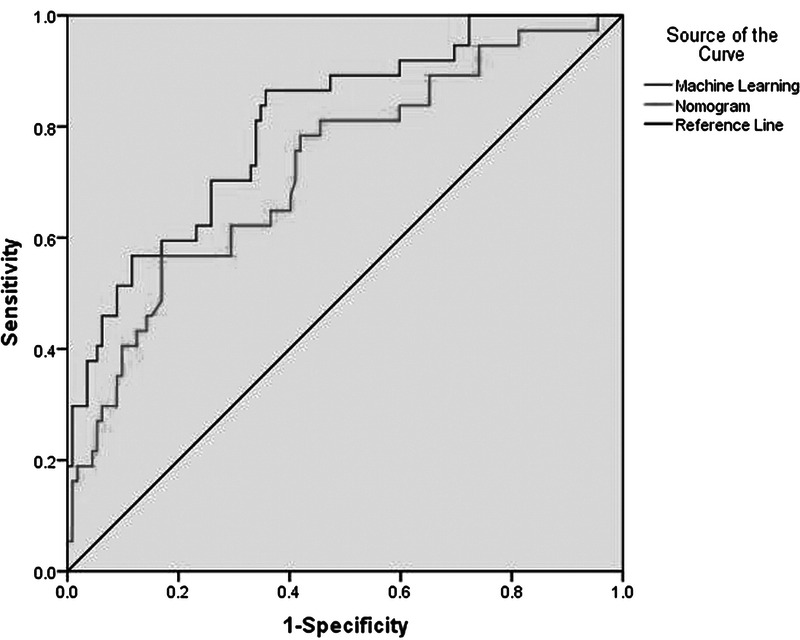
Receiver Operating Characteristic Curve for Surgical-Related Pressure Injuries by Machine Learning Model Versus Logistic Regression

By adopting the ML tool XGBoost, the developed pressure injury prediction model performed at an AUC value of 0.806. Figure 1 shows the receiver operating characteristic curve of the ML prediction model.

### Predictor Importance

Nine predictors entered the ML model. These predictors were patient age, gender, disease category, weight, duration of surgery, duration of cardiopulmonary bypass procedure, perioperative corticosteroid administration, use of intraoperative vasoactive agents, and use of postoperative vasoactive agents.

**Figure 2.**
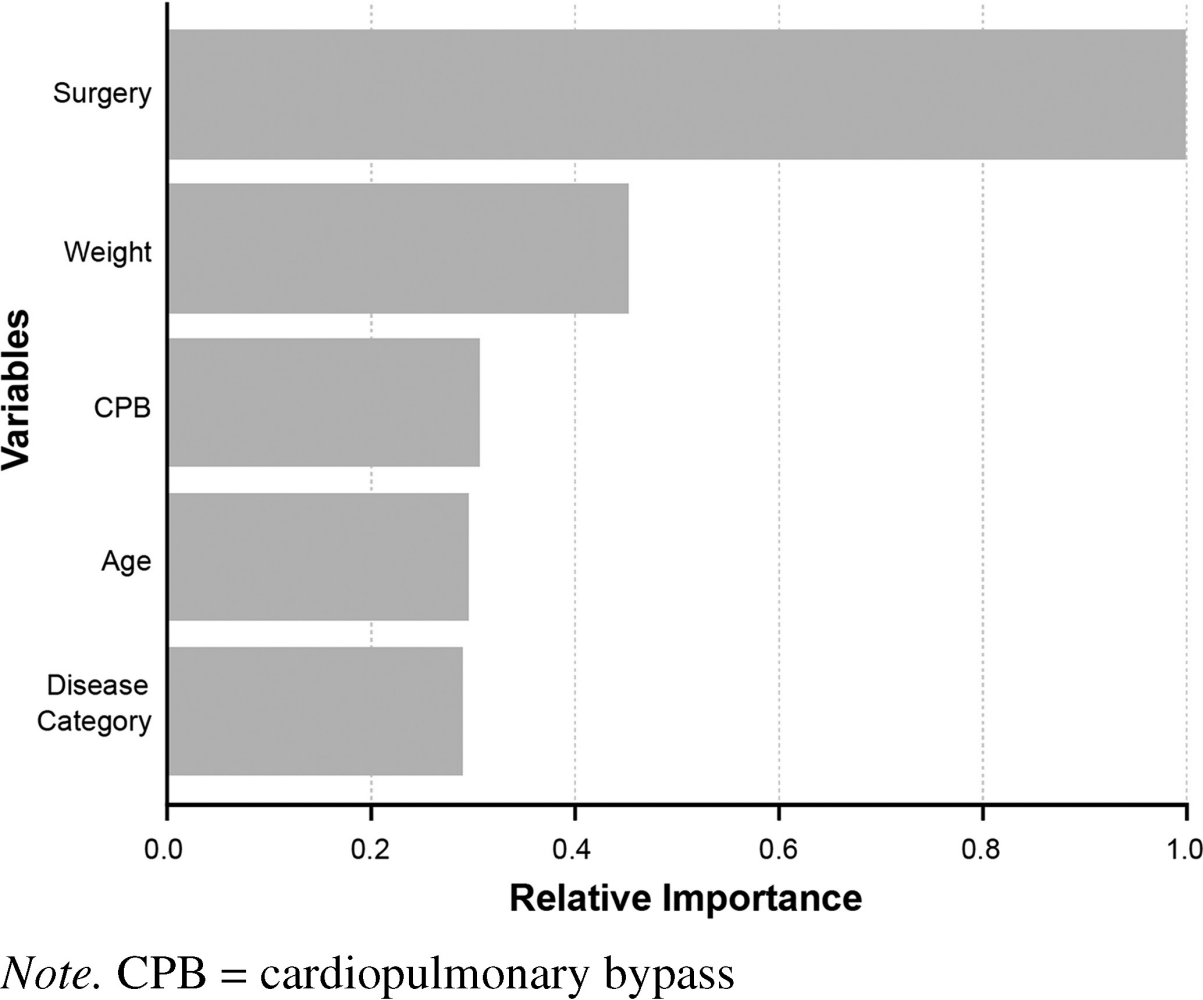
Relative Importance of Studied Variables to Surgical-Related Pressure Injuries

Finally, the importance metrics were aggregated to summarize the five predictors that were important in this ML model. These included, in rank order of decreasing importance, (a) duration of surgery (in minutes), (b) weight (in kilograms), (c) duration of cardiopulmonary bypass procedure (in minutes), (d) age (in years), and (e) disease category (e.g., congenital heart disease). The importance of these five risk factors in predicting SPRI was 0.426, 0.193, 0.131, 0.126, and 0.124, respectively. Findings indicate that duration of surgery was the most important risk factor for SRPI. The proportional importance of each input variable is shown in Figure 2. Risk factors of insignificant importance, including gender, perioperative corticosteroid administration, use of intraoperative vasoactive agents, and use of postoperative vasoactive agents, are not shown.

## Discussion

In this prospective observational study of surgical patients, we constructed a risk prediction model of SRPI development. The prediction model achieved an AUC of 0.806, indicating that this model has moderate predictive accuracy for SRPI in patients undergoing cardiovascular surgery.

In this study, an ML algorithm called XGBoost was adopted for feature selection and model construction. XGBoost is a nonparametric algorithm that does not assume a functional relationship between outcome and features, as is required for linear regression models (Jensen et al., 2012). As a result, this supervised ML method is a robust approach for handling correlated features. The resulting ML prediction model captured potentially powerful predictors of the development of SRPI. In this study, the highest risk of SRPI was borne by patients with longer surgery and cardiopulmonary bypass procedure durations, lower body weight, older age, and congenital heart disease.

Predictive models for SRPI have been developed previously. In a prior study conducted by the authors of this study, a nomogram score was constructed on the basis of logistic regression to predict the development of SRPI (Lu et al., 2017). The constructed logistic regression model contained four independent risk factors: duration of surgery, weight, disease category, and perioperative corticosteroid administration. The three risk factors duration of surgery, weight, and disease category were consistent with the ML model developed in this study. Moreover, age and duration of cardiopulmonary bypass procedure were also considered as important factors in the ML model. The logistic regression model was shown to have moderate power for predicting SRPI, with an AUC of 0.725, an outcome that was similar to this study. However, the ML model has more accurate discrimination power than the nomogram score. ML has been proposed by several authors as an approach to wound-tissue recognition. Kosmopoulos and Tzevelekou used ML for pressure injury diagnosis and presented some exploratory results (Kosmopoulos & Tzevelekou, 2007). Kaewprag et al. used the Bayesian network algorithm in ML to explore risk factors for pressure injuries in patients in intensive care units. That study indicated that the sensitivity of the ML predictive model was nearly three times higher than the logistic regression model, with no decline in overall accuracy (Kaewprag et al., 2017). Therefore, similar to logistic regression, ML may also be used as a technique along with data mining to improve assessment of risk of the development of SRPI.

The five most important variables based on the mean decrease in SRPI accuracy were, in descending order, duration of surgery, body weight, duration of cardiopulmonary bypass procedure, age, and disease category. A previous study (Chen et al., 2018) built an artificial neural network model to investigate the independent risk factors for SRPI in patients undergoing cardiovascular surgery. The factors identified included disease category, perioperative corticosteroid administration, age, and duration of surgery, and the importance of these factors to predicting SRPI was 0.268, 0.136, 0.237, and 0.360, respectively. In this study, two new risk factors, namely, weight and duration of cardiopulmonary bypass procedure, were identified. Duration of surgery is recognized as a high-risk factor for the development of SRPI. Extended duration of procedures leads to increased duration of hypoperfusion, ischemia of local compressed tissues, and decreasing temperature of the compressed position skin, which increase the risk of SRPI (Chen et al., 2017; O'Connell, 2106; Shen et al., 2015). According to a previous study, every 1-hour extension in surgery duration increases the risk of SRPI by 96% (Gao et al., 2018). In a meta-analysis of the association between duration of surgery and SRPI risk, the point estimates for surgery duration at 300 and 600 minutes, respectively, increased the SRPI risk by 3.653 and 13.344 times that of the risk at 60 minutes (Chen et al., 2017).

Weight was identified in this study as an important variable affecting SRPI risk. Although previous studies have shown that poor nutritional status is a common risk factor for pressure injury, only one previous study reported finding a correlation between higher risk of pressure injury and lower body mass in surgical patients (Gao et al., 2018). Most previous studies have cited body mass index as a potential risk factor and showed low body mass index as a significant predictor of SRPI (Alderden et al., 2018; Aloweni et al., 2019). In this study, we did not include body mass index, because it is not clear whether it is an independent risk factor for SRPI. Future studies may further confirm the predictive effect of body mass on SRPI.

The impact of cardiopulmonary bypass procedure duration was not adequately explored in a previous systematic review (Rao et al., 2016). In this study, duration of cardiopulmonary bypass procedure was deemed as one of the most significant of the examined variables. Our finding that patients who undergo cardiopulmonary bypass are more likely to develop SRPI agrees with other previous studies (Alderden et al., 2018; Gao et al., 2018). Moreover, SRPI experienced after cardiopulmonary bypass may be related to poor peripheral circulation perfusion caused by intraoperative hypothermia, utilization of a warming blanket after cardiopulmonary bypass, and/or lack of subcutaneous tissue protection at heel (Gao et al., 2018). Further studies should pay attention to the effective prevention of heel-related SRPI.

The variables that were found to have no significant predictive effect are also informative for future research. Corticosteroid is a common variable associated with SRPI in cardiovascular surgical patients, as the use of corticosteroid decreases the levels of growth factor, which is deemed as an important factor for pressure injury development (Feuchtinger et al., 2005; Wicke et al., 2000; Yang et al., 2013). However, in this study, corticosteroid administration was not found to be an important risk factor. Future researchers may take into account these factors and multidisciplinary wound care.

### Limitations

This study had limitations. First, the data were from a single healthcare institution within a confined geographic region. Thus, the generalizability of our findings may be limited. Second, not collecting data prospectively may affect the performance of the ML prediction model developed in this study. Third, the severity of all SRPI instances in this study were Stage 1.

### Conclusion

An ML model for predicting SRPI risk in cardiovascular surgical patients was constructed in this study. Integrating predictive analysis into clinical care holds the potential to better identify high-risk patients and provide appropriate predictive intervention. Future studies may build on these findings to develop a potentially more robust and sensitive ML model for predicting SRPI risk.
